# Predicting the potential distribution and climatic response of the endangered medicinal and edible species, *Anoectochilus roxburghii*, using an optimized MaxEnt model

**DOI:** 10.1038/s41598-025-24730-0

**Published:** 2025-11-20

**Authors:** Yaqin Hou, Liang Wu, Feng Jiang, Xingyue Fan, Dachen Luo, Xiaopeng Ai, Jing Wang, Ming Yang

**Affiliations:** 1 Department of Pharmacy, Affiliated Hospital of North Sichuan Medical College, Nanchong, 637000 Sichuan People’s Republic of China; 2 School of Pharmacy, North Sichuan Medical College, Nanchong, 637000 Sichuan People’s Republic of China; 3 Department of Pharmacy, The Second Clinical Medical College of North Sichuan Medical College, Nanchong, 637000 Sichuan People’s Republic of China; 4 Nanchong Key Laboratory of Individualized Drug Therapy, Nanchong, 637000 Sichuan People’s Republic of China; 5 School of Basic Medical Sciences, Wannan Medical College, Wuhu, 241000 Anhui People’s Republic of China; 6 Department of Respiratory and Critical Care Medicine, Affiliated Hospital of North Sichuan Medical College, Nanchong, 637000 Sichuan People’s Republic of China; 7 Department of Pharmacy, Xiamen Humanity Hospital, Xiamen, 361006 Fujian People’s Republic of China

**Keywords:** Climate change, Species distribution models, *Anoectochilus roxburghii*, Maxent, Environmental variables, Medicinal plants

## Abstract

Assessing how the habitat suitability and distribution of endangered species may alter under future climate scenarios is essential for robust conservation strategies. *Anoectochilus roxburghii*, an endangered and medicinally important orchid, faces significant threats from climate change due to its specific habitat requirements. This study predicts its potential distribution dynamics from the Last Glacial Maximum to the 2070s to inform conservation. We employed an optimized Maximum Entropy (MaxEnt) model, using species occurrence records and environmental variables to project its suitable habitats under past, current, and future (SSP126 and SSP585 scenarios) climates. The model performed excellently (AUC = 0.965), identifying the Mean Temperature of the Coldest Quarter (bio11) and Annual Precipitation (bio12) as the primary limiting factors. Historical simulations revealed habitat contraction into southern refugia during glacial periods and expansion during warmer interglacials. Future projections indicate a significant northward expansion of suitable habitats, a shift that is substantially more rapid and extensive under the high-emissions SSP585 scenario. These findings provide a scientific basis for proactive conservation. We recommend strengthening in situ protection in stable southern refugia, prioritizing germplasm collection from contracting populations, and guiding artificial cultivation into newly suitable northern regions to mitigate climate change impacts and reduce pressure on wild populations.

**Supplementary Information:**

The online version contains supplementary material available at 10.1038/s41598-025-24730-0.

## Introduction

Climate change is profoundly reshaping global ecosystems, altering species distributions, community structures, and ecosystem functions^1^. Rising temperatures, shifting precipitation patterns, and increasing frequency of extreme climatic events have disrupted the ecological niches of numerous plant species, leading to range contractions, upward or poleward migration, and even local extinctions^2^. For many rare and endangered plants, especially those with narrow ecological amplitudes and specific habitat requirements, climate-induced habitat loss poses an imminent threat to their survival^3,4^. More particularly, changes in climatic conditions can directly alter habitat suitability and resource availability, thereby reshaping species’ ecological niches and influencing their survival and persistence^5^. Thus, investigating how species respond to climate change is essential not only for predicting their future potential distributions but also for providing the critical scientific basis needed to develop effective strategies for germplasm resource management and species conservation.

*Anoectochilus roxburghii* (Wall.) Lindl., a perennial herb of the Orchidaceae family, is a highly prized plant distributed across several Asian countries, including China, Vietnam, India, Japan, and Thailand^6^. It serves as a quintessential dual-use species, widely utilized both as a functional food in soups and as a potent agent in traditional medicine^7^. For centuries, folk remedies have employed *A. roxburghii* to treat a wide array of ailments such as diabetes, hyperliposis, hepatitis, tumors, and hypertension^7^. Modern phytochemical investigations have validated this traditional wisdom, revealing a rich profile of bioactive compounds including polysaccharides, flavonoids, alkaloids, organic acids, and the notably active glycoside, kinsenoside^8,9^. These constituents underpin the plant’s broad spectrum of scientifically verified pharmacological activities, which include antidiabetic, hepatoprotective, antioxidant, anti-inflammatory, immunomodulatory, and antineoplastic effects^10–13^. Consequently, *A. roxburghii* commands significant market value and is increasingly sought after for incorporation into health products and pharmaceuticals.

Despite its immense medicinal and economic value, the survival of *A. roxburghii* is under severe threat. Unsustainable over-collection from the wild, coupled with its slow growth, low natural propagation rate, and specific habitat requirements, has critically depleted its natural populations^14^. In recognition of its precarious status, the species has been officially listed as a National Level II Protected Plant in China. The ecological niche of *A. roxburghii* is highly specialized. For example, research demonstrates that its growth, photosynthesis, and accumulation of medicinally important flavonoids are significantly dependent on precise light conditions, including both intensity (shade levels) and quality (wavelength)^15^. Furthermore, studies at the molecular level reveal a complex and sensitive response mechanism to abiotic stressors such as heavy metal contamination^16^. This demonstrated sensitivity to fine-scale environmental variables underscores the species’ narrow ecological amplitude, suggesting a limited capacity to buffer against environmental shifts. Consequently, this inherent vulnerability strongly suggests that global climate change, with its associated shifts in temperature and precipitation patterns, poses a profound threat to the stability and geographic range of the species’ suitable habitats. Therefore, predicting the potential distribution of *A. roxburghii* under future climate scenarios is of paramount importance for its long-term conservation.

To address this challenge scientifically, species distribution models (SDMs) have become powerful tools for predicting the potential suitable habitats of species by integrating known occurrence records with a suite of environmental variables^17,18^. Among the various algorithms available, the maximum entropy (MaxEnt) model is widely recognized for its high predictive performance and its ability to deliver robust results using only presence data^19–24^, making it particularly well-suited for studying rare and endangered species like *A. roxburghii*, for which the development of effective conservation strategies is of pressing urgency. By applying an optimized MaxEnt model in conjunction with future climate scenarios, it is possible not only to identify the key environmental drivers limiting the distribution of *A. roxburghii* but also to quantitatively forecast the expansion, contraction, and migration of its suitable habitats over time. These predictions hold profound practical significance, offering an evidence-based framework for identifying conservation priority areas, planning germplasm conservation for regions projected to become unsuitable, and guiding the artificial cultivation and sustainable management of this invaluable medicinal plant^25–27^.

In this study, we will evaluate the potential distribution of *A. roxburghii* under the current climate (1970–2000) and for two future periods (the 2050s: 2041–2060; the 2070s: 2061–2080) across two shared socioeconomic pathways (SSP126 and SSP585). Our objectives are as follows: (1) to apply an optimized MaxEnt model to predict the potential suitable areas and centroid migration trends of *A. roxburghii* under current and future scenarios, and to identify the dominant environmental factors limiting its distribution; (2) based on the analysis of its spatiotemporal dynamics, to identify stable (areas suitable in both current and future periods)highly suitable habitats and potential climate change refugia to provide scientific guidance for the species’ conservation. This study systematically examines the response of *A. roxburghii*'s potential suitable areas to global environmental change and aims to offer valuable recommendations for its priority conservation planning and management. Our findings contribute significantly not only to the restoration of wild populations of this endangered medicinal plant but also to the conservation of medicinal plant biodiversity by providing a targeted conservation framework for an endangered species.

## Results

### Model optimization and performance evaluation

**Fig. 1:**
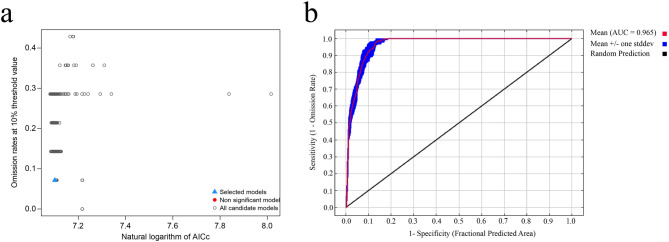
MaxEnt model optimization and performance evaluation results. (**a**) The results of the MaxEnt model optimization. The blue triangle indicates the selected optimal model, which has the lowest AICc value while maintaining a low omission rate. (**b**) The ROC curve for the optimized model. The red line represents the average result of 10 replicates, and the blue shaded area represents the standard deviation.

Following model evaluation, 23 met the low omission rate criterion (≤ 10%). Using a multi-criteria approach considering AICc, Delta AICc, and omission rate, the optimal model (M_4_F_lqt) was selected. This model minimized both AICc (1212.9) and Delta AICc (24.179) while maintaining a low omission rate of 0.071 (Fig. 1a, blue triangle). By contrast, the default-parameter model (M_1_F_lqpth) had higher AICc (1235.417), higher Delta AICc (46.696), and a higher omission rate (0.286), indicating that parameter optimization substantially improved model performance. To further evaluate the model’s predictive capability, multiple complementary performance metrics were employed. A Receiver Operating Characteristic (ROC) analysis yielded an average Area Under the Curve (AUC) value of 0.965 ± 0.005, exceeds the 0.8 threshold (Fig. 1b), confirming the model’s discriminative ability. Collectively, these results demonstrate that the optimized MaxEnt model exhibits excellent predictive performance and stability for estimating the potential distribution of *A. roxburghii*.

### The effects of key environmental variables on the distribution of *A. roxburghii*

**Fig. 2:**
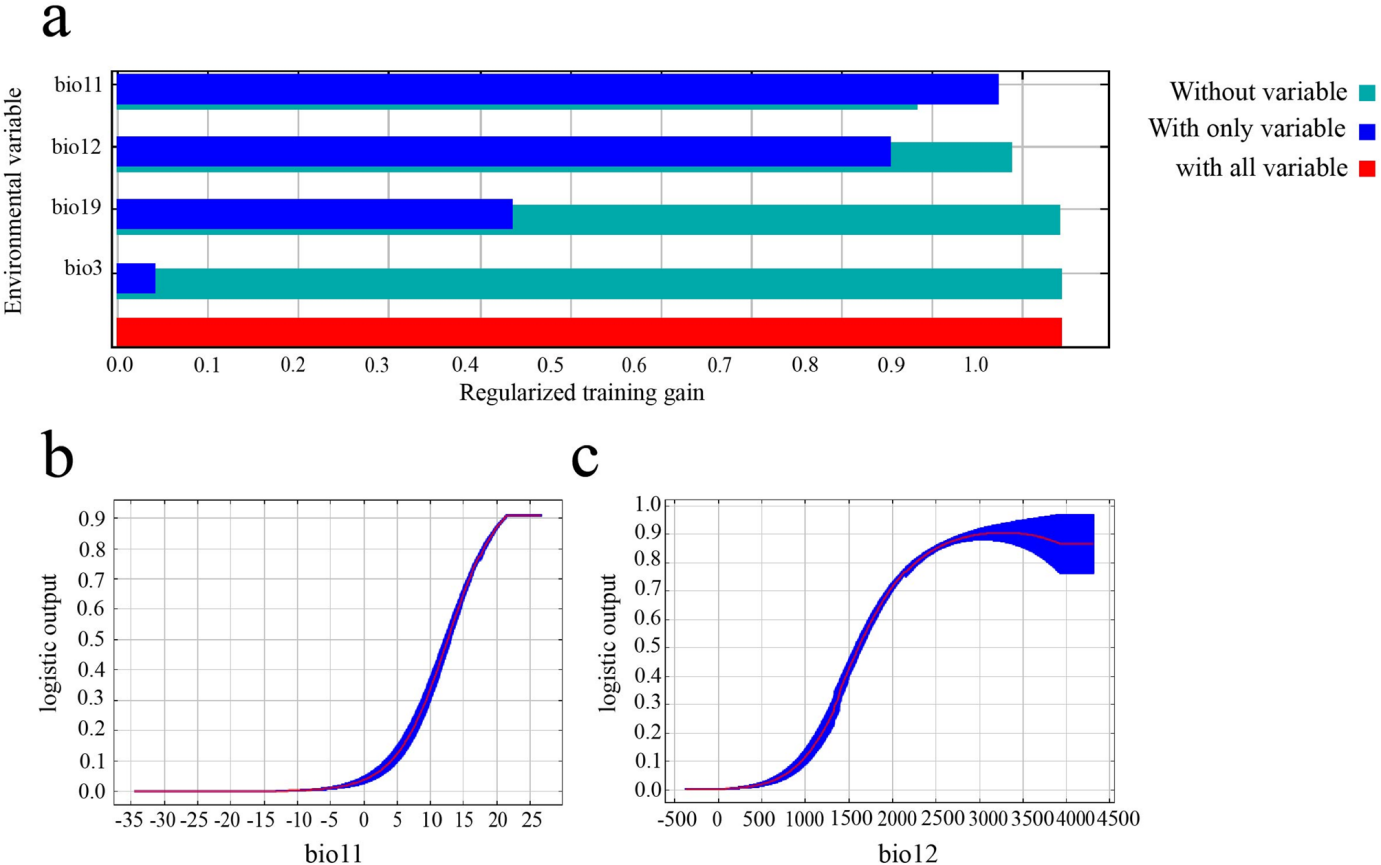
Jackknife test of environmental variable importance and response curves for key variables in the MaxEnt model. (**a**) The results of the Jackknife test evaluating the relative importance of environmental variables. (**b**) Response curves illustrating the relationship between habitat suitability (logistic output) and bio11. (**c**) Response curves illustrating the relationship between habitat suitability (logistic output) and bio12.

The analysis of percent contribution and permutation importance revealed that the Mean Temperature of the Coldest Quarter (bio11) and Annual Precipitation (bio12) are the primary determinants of *A. roxburghii*'s distribution, collectively accounting for 94.5% of the percent contribution and 99.9% of the permutation importance (Table 1). To complement these results, the Jackknife test was further applied to visualize the independent explanatory power of each environmental variable. This test evaluates the change in model gain when each variable is used in isolation or omitted, thereby identifying variables that contain unique information not shared by others. Among all environmental factors, omitting the Mean Temperature of the Coldest Quarter (bio11) led to the largest decrease in model gain, demonstrating that this factor contributes the most unique information that cannot be replaced by other variables (Fig. 2a). Based on our model, winter temperature and annual precipitation were identified as the most significant environmental factors influencing the potential distribution of *A. roxburghii*, with the species’ occurrence probability varying significantly in response to these conditions. The response curve for bio11 indicates that habitat suitability increases sharply as the mean temperature of the coldest quarter rises above 5 °C, reaching optimal conditions at approximately 20 °C and above (Fig. 2b). For bio12, the species shows a strong preference for high rainfall environments, with suitability increasing significantly in areas receiving over 1500 mm of annual precipitation and peaking at approximately 3500 mm before slightly declining (Fig. 2c).

**Table 1:** Percentage contribution and permutation importance of environmental factors used for modeling.

Variable	Description	Percent contribution	Permutation importance
bio11	Mean temperature of coldest quarter (℃)	49.7	88.5
bio12	Annual precipitation (mm)	44.8	11.4
bio19	Precipitation of coldest quarter (mm)	5.1	0
bio3	Isothermality (BIO2/BIO7) (× 100)	0.4	0

### Potential suitable habitat distribution of *A. roxburghii* under paleo-climatic scenarios

**Fig. 3:**
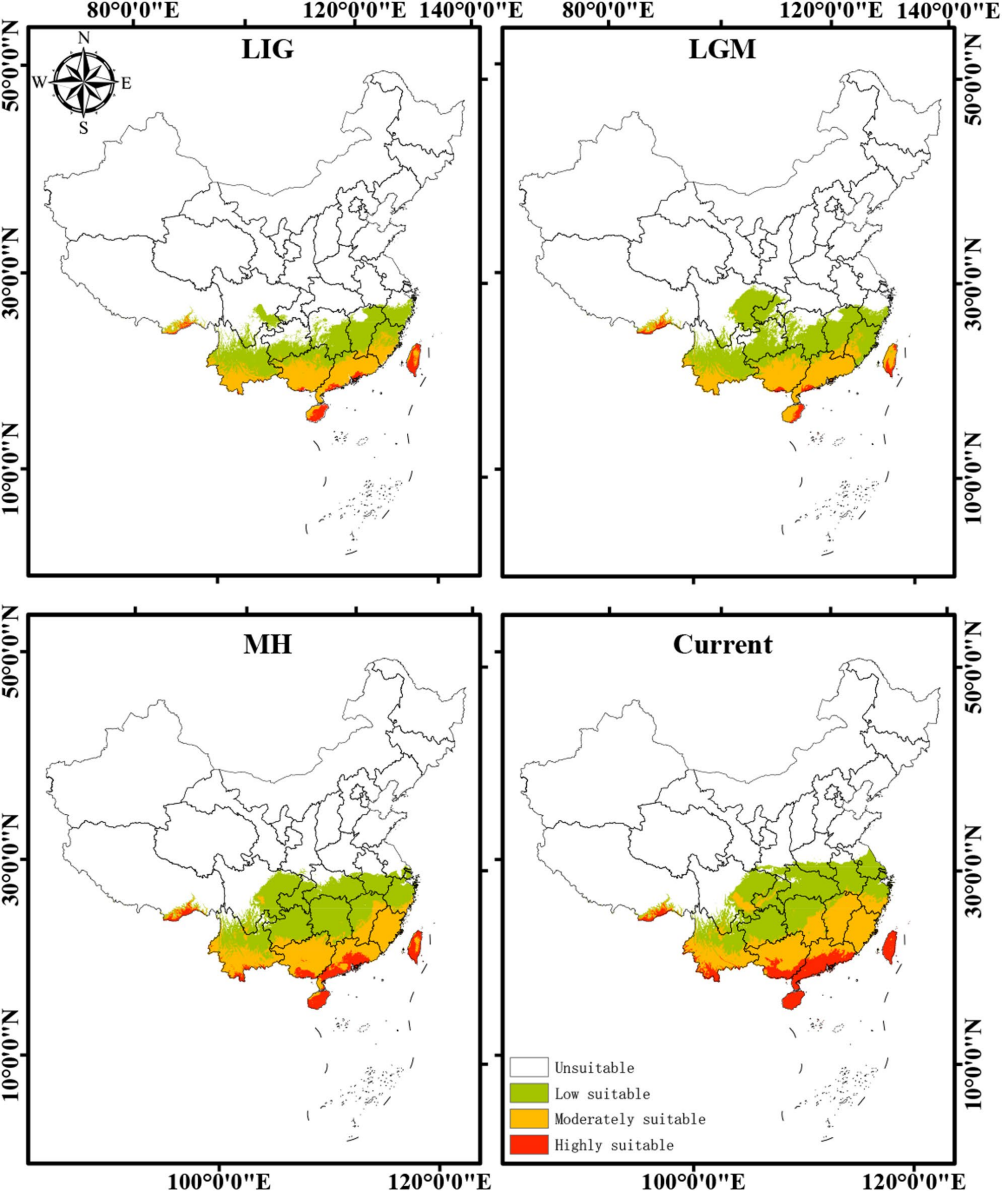
Potential suitable habitat distribution of *A. roxburghii* under current and future scenarios. The colors in the legend represent different suitability levels, from low suitability (green) to high suitability (red). LIG: Last Interglacial; LGM: Last Glacial Maximum; MH: Mid-Holocene.

The potential distribution of *A. roxburghii* under historical climatic conditions, including the Last Interglacial (LIG), Last Glacial Maximum (LGM), and Mid-Holocene (MH), was projected to reconstruct its historical biogeographical patterns (Fig. 3). During the LIG (126.91 × 10^4^ km^2^), the species’ range was relatively broad, with low-suitability habitats dominating (59.2%), moderately suitable areas contributing 35.5%, and highly suitable habitats accounting for 5.3%. The distribution extended northward into the Yangtze River Basin, indicating a warm-adapted expansion. In contrast, during the colder LGM (148.35 × 10^4^ km^2^), the suitable area contracted and shifted southward. Low-suitability habitats further dominated (64.9%), while highly suitable areas declined sharply to only 3.2%. The remaining suitable habitats were mainly confined to refugia in southern coastal regions, including Guangdong, Guangxi, Hainan Island, and Taiwan Island. Following the glacial retreat, the MH (199.07 × 10^4^ km^2^) witnessed a pronounced re-expansion of suitable habitats. Moderate-suitability areas became dominant (35.1%), while highly suitable habitats increased to 8.5%, reflecting a pattern broadly similar to the present distribution but slightly more fragmented. At present (222.21 × 10^4^ km^2^), the distribution is more extensive than in all three historical periods, with low- (50.5%), moderate- (37.5%), and high-suitability (12.1%) habitats jointly shaping a northward-shifted and relatively continuous range (Table S1). Overall, these dynamics illustrate a consistent pattern of “glacial contraction–interglacial expansion”, highlighting the species’ sensitivity to long-term climate fluctuations and its strong dependence on warm–humid conditions.

### The suitable habitat distribution of *A. roxburghii* under future climate change

**Fig. 4:**
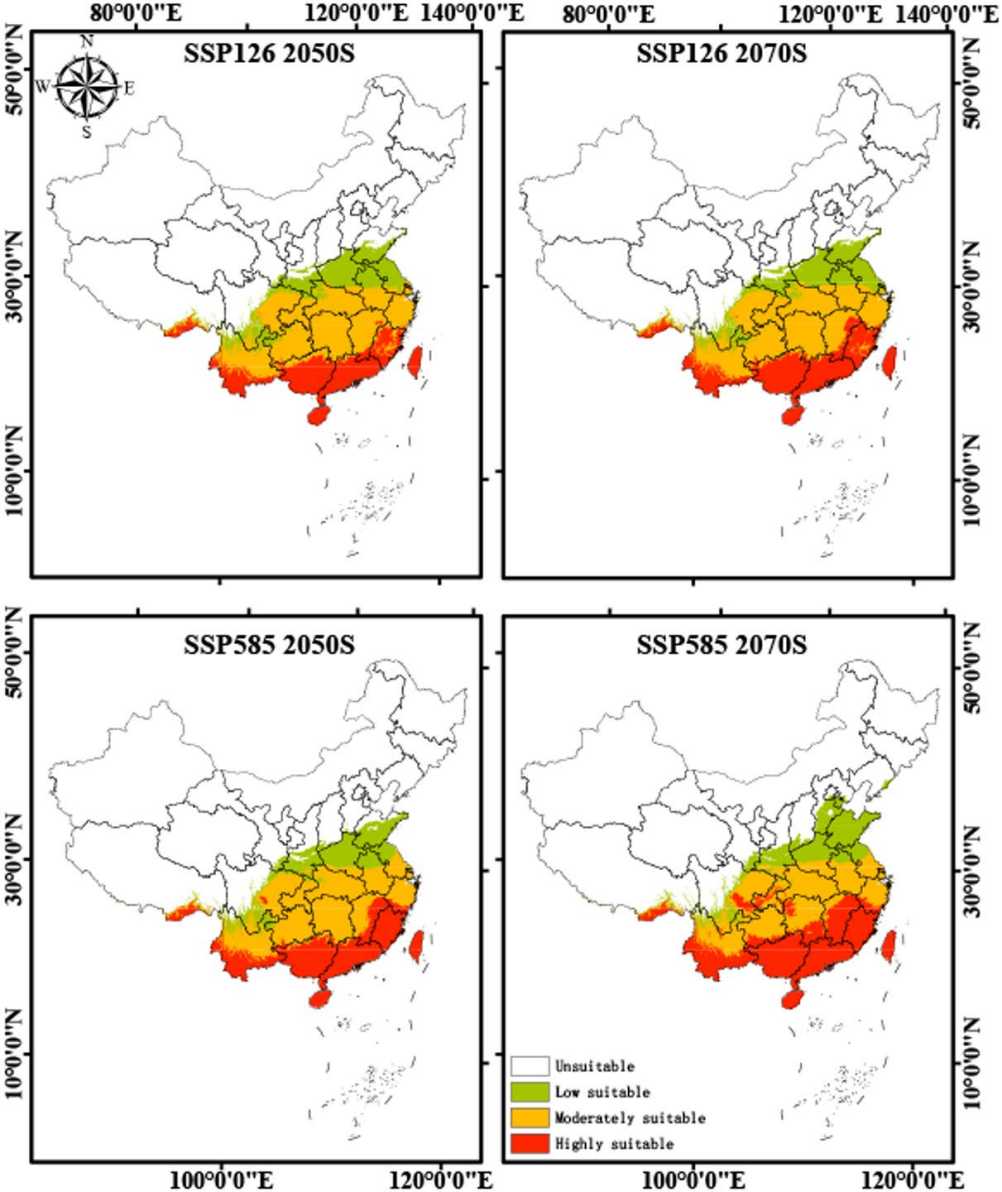
Potential suitable habitat distribution of *A. roxburghii* under current and future climatic scenarios. The colors in the legend represent different suitability levels, from low suitability (green) to high suitability (red).

The potential distribution of *A. roxburghii* under future climate change was projected for two future periods (2050s and 2070s) under two different Shared Socioeconomic Pathways (SSP126 and SSP585), and the results are presented in Fig. 4. As shown in Fig. 4, the projected suitable habitat area is expected to increase by 36.9% under the SSP585 scenario by the 2070s, with a general trend of migration towards higher latitudes. However, the core distribution area remains concentrated in Southern and Southeastern China. Specially, under the low-emission scenario (SSP126), the total suitable habitat of *A. roxburghii* expands from 222.2 × 10^4^ km^2^ at present to 258.5 × 10^4^ km^2^ in the 2050s and 261.3 × 10^4^ km^2^ in the 2070s. Within this expansion, the proportion of highly suitable habitats increases from 12.1% at present to 24.7% in the 2050s, while moderately suitable areas also expand from 37.5% to 51.1%. In contrast, low-suitability habitats decline markedly from 50.5% to 24.3%. By the 2070s, the proportions remain generally stable, with only slight adjustments across categories. Under the high-emission scenario (SSP585), the total suitable area increases to 268.0 × 10^4^ km^2^ in the 2050s and further to 289.2 × 10^4^ km^2^ in the 2070s. The changes in suitability classes are more pronounced: highly suitable habitats increase to 29.8% in the 2050s and further to 36.9% in the 2070s, while moderately suitable areas first rise to 47.7% before declining slightly to 39.4%. Meanwhile, low-suitability habitats contract sharply, accounting for only about 22–24% of the total area. These results reveal a consistent trend of decreasing low-suitability areas and increasing highly suitable areas under both scenarios, with more significant shifts under SSP585 (Table S1). These results suggest that ongoing climate warming will consistently drive *A. roxburghii* to seek new suitable environments at higher latitudes.

### Spatiotemporal dynamics of suitable habitats for *A. roxburghii* under future climate scenarios

**Fig. 5:**
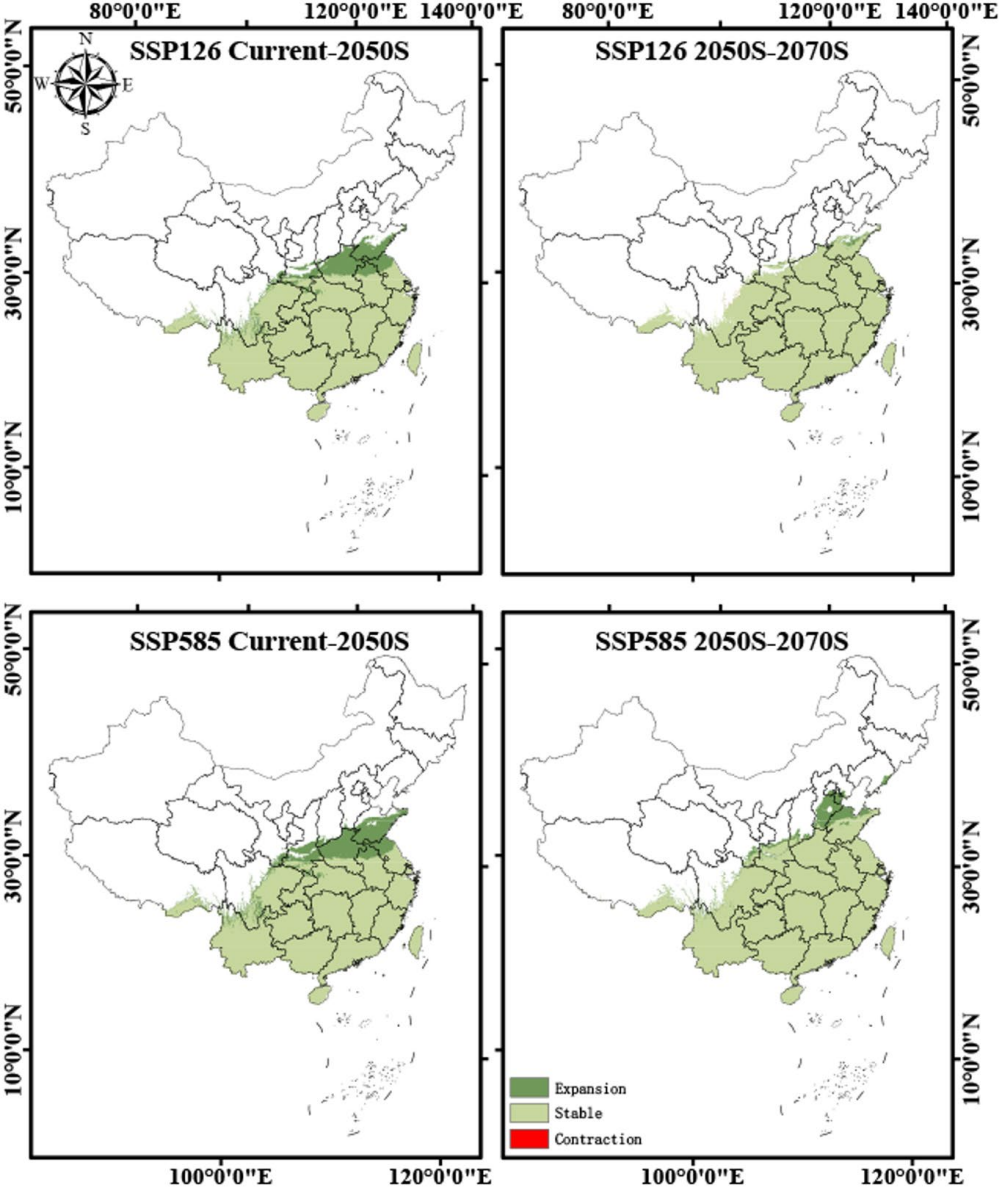
Spatiotemporal dynamics of suitable habitats for *A. roxburghii* under future climate scenarios. Dark green indicates areas of expansion, light green indicates stable habitats, and red indicates areas of contraction.

To further reveal the spatiotemporal dynamics of the suitable habitat for *A. roxburghii* under future climate change, this study analyzed the changes (expansion, contraction, and stability) between the current and future periods, as well as between different future periods (Fig. 5). The analysis indicates that most of the core suitable habitat for *A. roxburghii* will remain stable, primarily concentrated in the southern Chinese provinces of Fujian, Yunnan, Guangxi, Guangdong, and Guizhou. These regions are expected to serve as persistent highly suitable habitats under both SSP126 and SSP585 scenarios. All scenarios predict a significant expansion of suitable habitats, which is the dominant trend. The expansion zones are consistently located at the northern edge of the current distribution, extending into higher-latitude areas such as Hubei, Anhui, Jiangsu, and southern Henan. This northward expansion is more pronounced and extensive under the high-emissions scenario (SSP585) compared to the low-emissions scenario (SSP126). Quantitatively, from the current period to the 2050s, the total suitable habitat of *A. roxburghii* is projected to increase by 16.34% under SSP126 and 20.61% under SSP585. Within this expansion, highly suitable areas show the most dramatic growth (+ 138.09% under SSP126 and + 198.54% under SSP585), moderately suitable areas also expand (+ 58.60% and + 53.42%), whereas low-suitability habitats decline sharply (− 44.07% and − 46.20%). From the 2050s to the 2070s, the overall expansion slows, with only a slight increase under SSP126 (+ 1.07%) but a continued rise under SSP585 (+ 7.91%). During this period, highly suitable habitats continue to expand (+ 13.9% and + 33.5%), moderately suitable areas show modest declines (− 5.45% and − 10.84%), and low-suitability habitats record minor gains (+ 1.67% and + 13.73%). In contrast, habitat contraction is projected to be minimal and sporadic. The area of contraction is significantly smaller than the area of expansion in all scenarios (Table S1). Overall, the potential distribution of *A. roxburghii* shows a clear dynamic pattern of northward migration, with habitat gains at its northern boundary substantially outweighing minor habitat losses at its southern edge.

### Migration routes of the core suitable habitat centroid

**Fig. 6:**
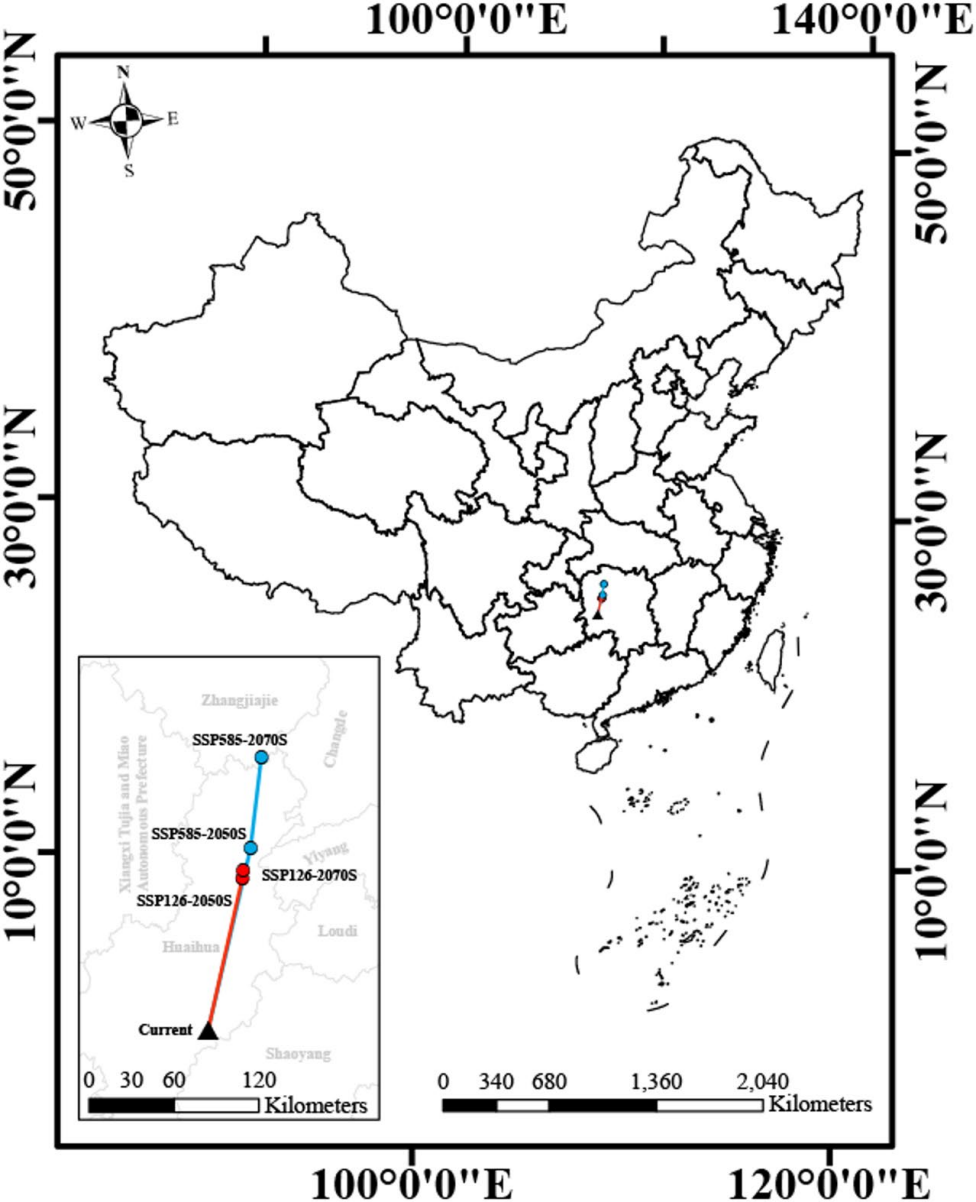
Migration routes of the suitable habitat centroid for *A. roxburghii* under future climate scenarios. The black triangle indicates the current centroid location; the red and blue paths represent the migration routes under the low-emissions (SSP126) and high-emissions (SSP585) scenarios, respectively.

To quantify the directional shift of *A. roxburghii*'s highly suitable habitats in response to climate change, we calculated the geographic centroid of the highly suitable areas for the current and future periods (Fig. 6). The results reveal a clear and consistent northward migration trend for the species’ highly suitable habitat under both climate scenarios. Under the low-emissions scenario (SSP126), the centroid is projected to migrate progressively northward from its current location, with the total displacement reaching approximately 140 km by the 2070s. In contrast, the high-emissions scenario (SSP585) predicts a much more substantial and accelerated migration. The centroid is forecasted to shift a greater distance northward by the 2050s and continue this trajectory, resulting in a northward shift of the highly suitable habitat centroid of approximately 280 km by the 2070s, nearly double the distance predicted under the SSP126 scenario. It should be noted that this centroid shift represents a spatial displacement of the predicted suitable habitat, not necessarily the realized migration of populations. The actual capacity of *A. roxburghii* to track this niche shift will depend on its dispersal ability, habitat connectivity, and ecological constraints. Nevertheless, the magnitude of this projected displacement highlights the strong influence of climate warming on the redistribution of the species’ potential niche in geographical space.

## Discussion

By optimizing the MaxEnt model parameters, we achieved excellent predictive performance for the potential distribution of *A. roxburghii*, as indicated by an average AUC value of 0.965. This result underscores the reliability of using an optimized model to explore the ecological niche of this endangered species, a conclusion consistent with other recent studies on rare plants^28,29^. Our analysis identified the Mean Temperature of the Coldest Quarter (bio11) and Annual Precipitation (bio12) as the two most critical environmental factors shaping the distribution of *A. roxburghii*, collectively contributing to over 90% of the model’s predictive power. The overwhelming importance of bio11 (Permutation importance: 88.5%) highlights that winter temperature is the primary limiting factor for the survival of this species. The response curve shows that habitat suitability drops sharply when the mean temperature of the coldest quarter falls below 5 °C, which is consistent with its natural distribution in the subtropical and tropical regions of Southern China, where frost is rare. This finding is further supported by literature describing *A. roxburghii*'s sensitivity to environmental stress and its specific habitat requirements in warm, humid forest understories, where optimal growing temperatures range from 18 to 25°C^30^. The strong dependence on Annual Precipitation (bio12), with an optimal range around 3500 mm, also corresponds to its known preference for moist environments. These quantified thresholds provide a clear ecological explanation for its current geographic range and its vulnerability to climatic shifts.

The reconstruction of *A. roxburghii*'s distribution under paleo-climatic conditions reveals a classic pattern of range dynamics in response to glacial-interglacial cycles. During the LGM period, its suitable habitat significantly contracted and retreated to southern refugia, primarily in coastal South China and on the islands of Hainan and Taiwan. This “glacial contraction” is a common biogeographical pattern for subtropical species in East Asia^31–33^. Conversely, during the warmer and wetter LIG and MH periods, the species experienced significant “interglacial expansion”, with its range extending northward. This historical sensitivity strongly suggests that *A. roxburghii* will respond dynamically to future anthropogenic climate change. Our future projections consistently show that the total suitable area for *A. roxburghii* will expand, driven by a distinct northward migration. This projected northward shift represents a common response pattern for species under climate warming, as they track the geographic location of their climatic niche^34^. According to our findings, winter temperature (mean temperature of the coldest quarter, bio11) is the primary climatic factor limiting the distribution of *A. roxburghii*. Hence, the projected rise in winter temperatures will likely unlock new, previously unsuitable habitats at higher latitudes by alleviating low-temperature stress, creating opportunities for the species’ expansion. The critical role of temperature in shaping the distribution of specialized plant species has been well-documented, especially for those limited by thermal conditions^35,36^. The magnitude of this shift is directly linked to the emissions scenario; the high-emissions SSP585 scenario resulted in a more rapid and extensive northward expansion and a nearly doubled centroid migration distance compared to the low-emissions SSP126 scenario. This indicates that unchecked climate change could dramatically accelerate the reshaping of this species’ geographic range. Such rapid, climate-induced shifts in distribution pose significant challenges to ecosystem stability and the conservation of endangered species^37^.

The findings of this study provide a spatially explicit and forward-looking scientific basis for the conservation and management of the endangered and medicinally important *A. roxburghii*. Based on our predictions, the following strategies are proposed: (1) Strengthening in situ conservation in highly suitable habitats. Our models identify a large, stable highly suitable habitat across future scenarios, concentrated in the provinces of Fujian, Guangdong, Guangxi, Yunnan, and Guizhou. These regions represent long-term climate refugia and should be designated as the highest priority for in situ conservation. A gap analysis of existing nature reserves within these stable zones is recommended to ensure they adequately protect the most suitable areas for *A. roxburghii*. (2) Urgent germplasm collection in contracting areas. Although limited, habitat contraction is predicted at the southern and western edges of the current distribution. These peripheral populations may possess unique genetic adaptations but are at the greatest risk of extirpation due to climate change. Therefore, it is crucial to prioritize these areas for ex situ germplasm conservation to preserve the species’ full genetic diversity. (3) Guiding artificial cultivation and assisted migration. The predicted emergence of new suitable habitats further north, particularly in Hubei, Anhui, and southern Henan under the SSP585 scenario, offers valuable guidance for artificial cultivation. Establishing cultivation bases in these regions, which are projected to be suitable in the future, could help meet the high market demand for *A. roxburghii* while simultaneously reducing the harvesting pressure on vulnerable wild populations.

Despite the robust performance of our model, several limitations should be acknowledged. The accuracy of any SDM is fundamentally dependent on the quality and spatial resolution of the input data. While we employed a rigorous data cleaning protocol, potential biases in species occurrence records from sources like GBIF are unavoidable^38^. Furthermore, this study models the species’ potential climatic niche, but does not account for other factors like biotic interactions (e.g., pollinators, competitors), soil properties, or human land use, which can also constrain its actual distribution^39^. Finally, our projections of future range shifts assume that *A. roxburghii* can disperse to and colonize all newly suitable areas. In reality, the species’ actual ability to track its shifting climate niche will be constrained by its dispersal capacity, a factor that requires further field investigation^40^. Therefore, the projected expansions should be interpreted as potential future habitats rather than guaranteed future ranges.

## Materials and methods

### Species occurrence data collection

The geographical distribution data for *A. roxburghii* in this study were compiled from the Global Biodiversity Information Facility (GBIF) and the Chinese Virtual Herbarium (CVH) (Table S2). GBIF records were accessed on 15 July 2025 via GBIF.org, GBIF Occurrence Download (10.15468/dl.6um4gz). To enhance the integrity of the occurrence data, a strict filtering protocol was applied to remove all entries that were duplicated, incomplete, or possessed erroneous geographic coordinates. Specifically, this includes: (1) removal of duplicate records; (2) removal of records with incomplete or missing coordinates; and (3) using the CoordinateCleaner package in R to flag and remove records that were geographically unlikely (e.g., located in the sea, at country centroids, or far outside the species’ known native range). To minimize the influence of spatial clustering and non-random sampling in the dataset, the occurrence points were spatially filtered at a resolution of 2.5 arc-minutes (approx. 5 km × 5 km) using the “Spatially Rarefy Occurrence Data for SDMs” tool in SDMtoolbox v2.6 (ArcGIS 10.8)^41^, ensuring that only one record was retained per grid cell. The 2.5 arc-minute (~ 5 km) resolution was chosen as a trade-off between ecological detail and computational feasibility. Critically, many paleo-climatic datasets used in this study are not available at finer resolutions, so using 2.5 arc-minutes allowed for consistency across all time periods analyzed. After this process, a total of 55 valid geographical distribution records were obtained and saved in CSV format for subsequent modeling (Figure S1).

### Environmental variables acquisition

The environmental variables used in this study were retrived from the WorldClim database^42^. Current climate data (1970–2000), comprising 19 bioclimatic variables (Table S3), were obtained from the WorldClim database at a spatial resolution of 2.5 arc-minutes (Table S4). To minimize any potential temporal mismatch between occurrence records and the climatic data (1970–2000), we filtered the dataset to exclude any records collected before 1970. Paleoclimate data for the MH (~ 6,000 years BP) and the LGM (~ 22,000 years BP) were derived from the CCSM4 model, while data for the Last Interglacial (LIG; ~ 120,000–140,000 years BP) were resampled to a resolution of 2.5 arc-minutes using ArcGIS. Future climate data were sourced from the BCC-CSM2-MR climate model under the Coupled Model Intercomparison Project Phase 6 (CMIP6)^43^. The CCSM4 (for paleo-climate) and BCC-CSM2-MR (for future climate) GCMs were selected because they are well-established, widely used models that have shown good performance in simulating climate patterns in our study region (East Asia)^44,45^. To assess the impacts of different emission pathways, two Shared Socioeconomic Pathways were selected: SSP126, representing a low-emission, sustainable development pathway, and SSP585, representing a high-emission, fossil fuel-driven pathway^46^. The projections cover two future periods: the 2050s (2041–2060) and the 2070s (2061–2080). To reduce multicollinearity and ensure the objectivity of environmental variable selection, a two-step filtering process was employed. First, a preliminary MaxEnt model was run incorporating all 19 bioclimatic variables under default settings (maximum 500 iterations). Variables with a percent contribution of 0% were excluded, taking advantage of the model’s intrinsic L1 regularization, which penalizes and effectively eliminates predictors with negligible explanatory power. Subsequently, a Pearson correlation analysis was performed on the environmental variables using SPSS statistics 26.0 software (Figure S2). For any pair of variables with a high correlation coefficient (|r|> 0.7), the variable with the lower contribution or less biological significance was removed. Further analysis was performed using the R package ‘usdm’ (version 2.1–7) to conduct VIF (Variance inflation factor) analysis on the retained environmental variables (bio3, bio11, bio12, and bio19) to ensure multicollinearity was minimized.

### MaxEnt model optimization and construction

Model parameter optimization was performed using the R package kuenm v 1.1.10 to identify the best model configuration^47^. A total of 248 candidate models were generated by testing a range of regularization multiplier (RM) values (0.5 to 3 in 0.5 increments, plus 4 and 5) across 31 feature class combinations. The optimal model was selected based on the lowest corrected AICc score^48^, while also considering statistical significance, as indicated by the partial ROC test implemented in the kuenm package, which confirmed predictive performance significantly better than random, and a low omission rate (< 10%). The final model was constructed in MaxEnt v.3.4.3 using the optimized parameters^49^. The occurrence data were randomly partitioned, with 75% used for model training and 25% for testing. To ensure model stability, the process was replicated 10 times using the bootstrap method, and the average result was used. The output format was set to logistic, which provides suitability values ranging from 0 to 1, representing the probability of species presence.

### Model evaluation and habitat suitability classification

Model performance was evaluated using the AUC of the ROC plot^50^. An AUC value > 0.9 indicates excellent performance, while > 0.8 is considered good. Then, the Jackknife test was used to assess the relative importance and independent contribution of each environmental variable to the model’s predictive power. To visualize predicted habitat suitability from MaxEnt, the continuous probability raster was reclassified in ArcGIS 10.8 into four categories. Thresholds were determined using three approaches: (1) the Balance Training Omission, Predicted Area, and Threshold Value method^51^ (BTPT = 0.0474), (2) the Maximum Training Sensitivity plus Specificity method 12^51^ (MTSS = 0.1702), and (3) the Natural Breaks (Jenks) method^52^ (0.5034). The crucial threshold separating suitable from unsuitable areas was determined by the 'Maximum Training Sensitivity Plus Specificity’ (MTSS) logistic threshold Based on these thresholds, the predicted habitat suitability was categorized into four levels: Unsuitable (*p* < 0.0474), Lowly Suitable (0.0474 ≤ *p* < 0.1702), Moderately Suitable (0.1702 ≤ *p* < 0.5054), and Highly Suitable (*p* ≥ 0.5054).

### Analysis of suitable area dynamics and centroid migration

To analyze the impact of climate change on the suitable habitats of *A. roxburghii* under different emission scenarios, spatial analysis tools in ArcGIS were used to conduct an overlay analysis of the current and future distribution maps. A spatial overlay of the habitat suitability maps was performed to delineate the dynamics of the species’ range, categorizing changes into zones of expansion, contraction, and stability, after which the area of each category was quantified. Furthermore, the centroid migration of the highly suitable habitat was analyzed using the Mean Center tool in SDMToolbox. To elucidate the migratory response of the species’ highly suitable habitat to climate change, we first determined the geographic mean center of the highly suitable areas for each time period. Subsequently, we mapped the trajectory, direction, and distance of these centroid shifts to quantify the overall trend.

## Supplementary Information

Supplementary Information 1.

Supplementary Information 2.

Supplementary Information 3.

Supplementary Information 4.

Supplementary Information 5.

Supplementary Information 6.

## Data Availability

Data are contained within the article and Supplementary Materials.
