# Supplementary figures and images for: Predicting the potential distribution and climatic response of the endangered medicinal and edible species, Anoectochilus roxburghii, using an optimized MaxEnt model
Source: Sci Rep. 2025 Nov 20;15:40978. doi: 10.1038/s41598-025-24730-0 (PMC12635229; doi:10.1038/s41598-025-24730-0)

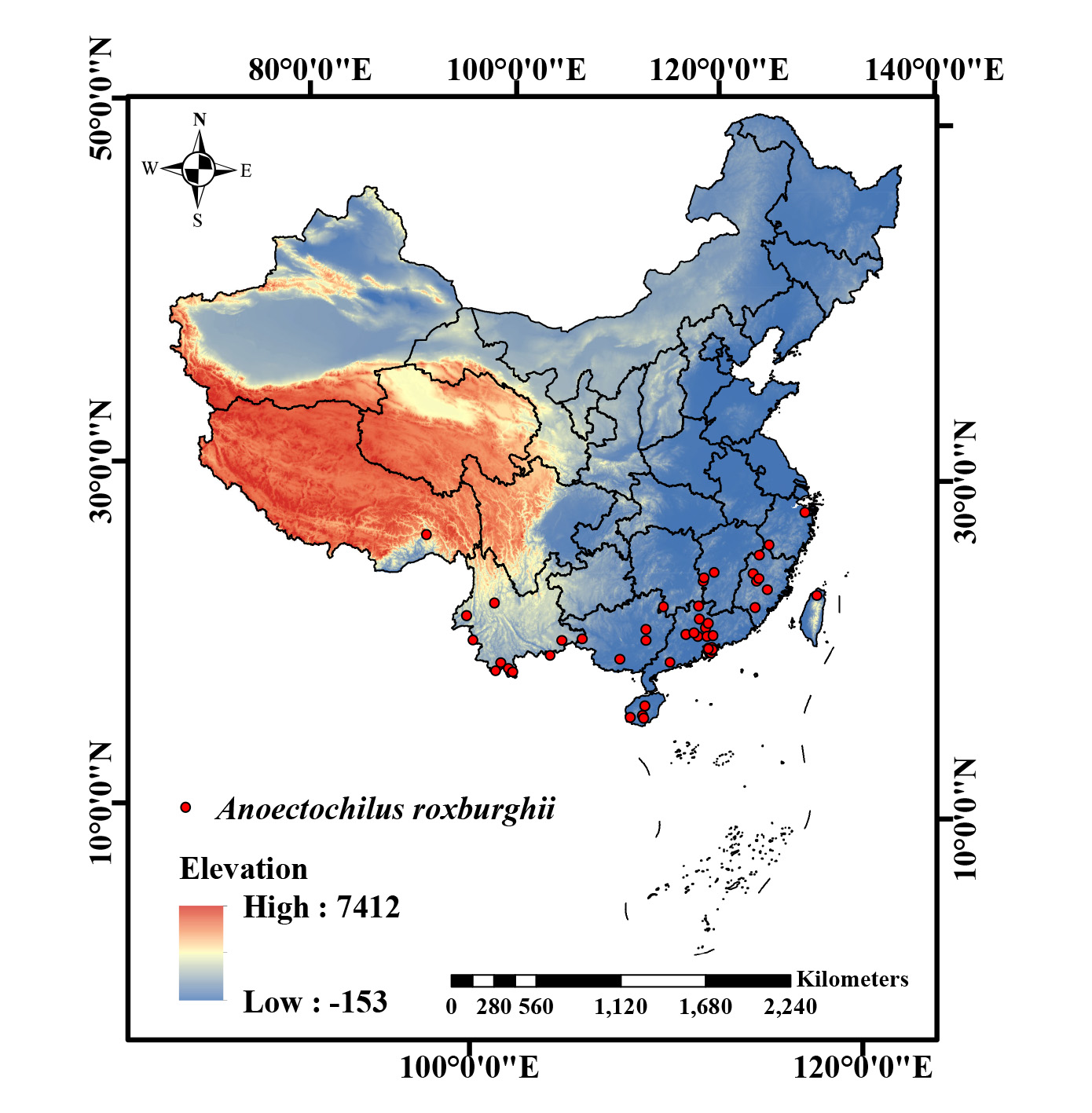

Supplement: Supplementary file 1 — Supplementary Information 1. [file 41598_2025_24730_MOESM1_ESM.jpg]

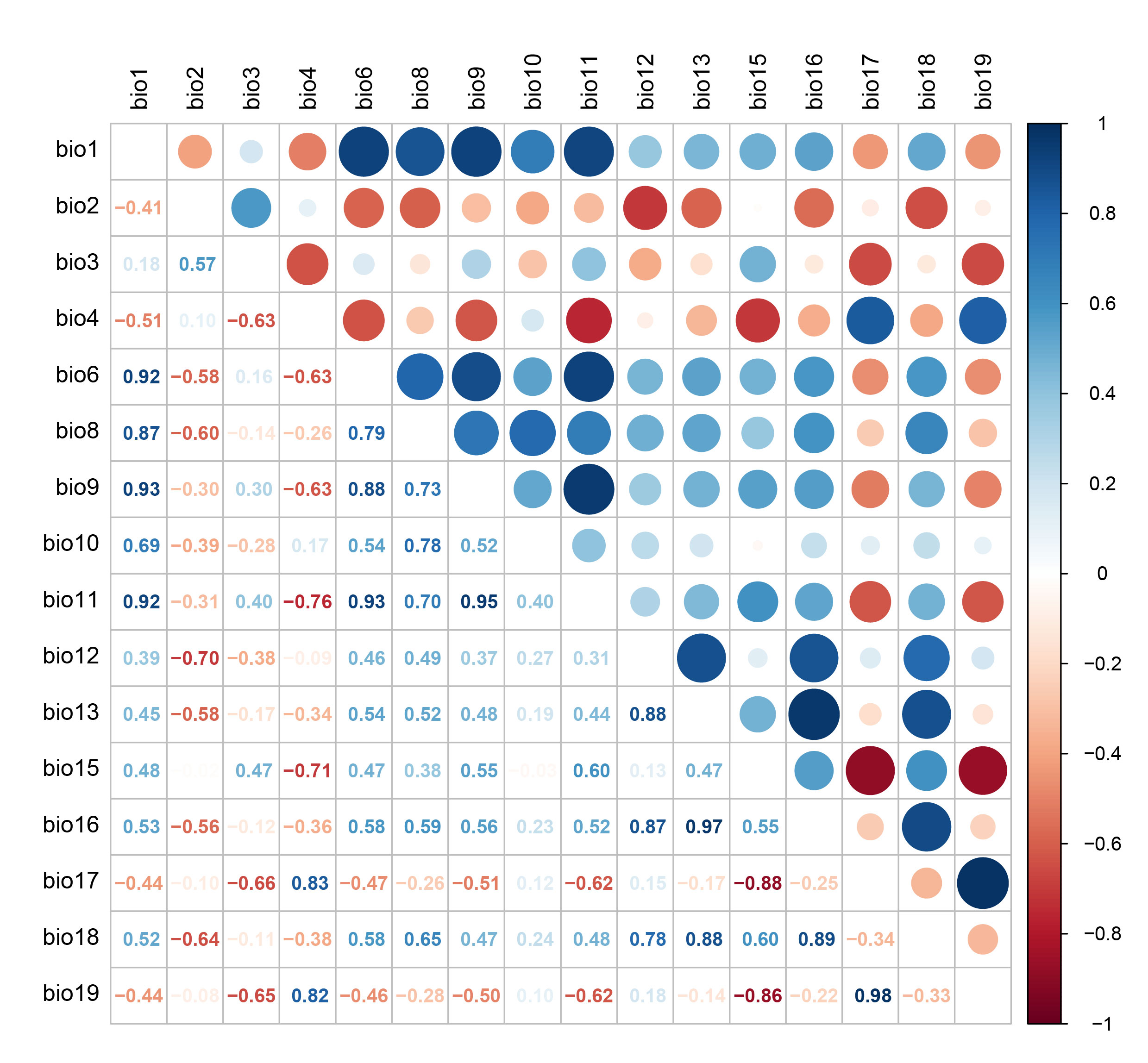

Supplement: Supplementary file 2 — Supplementary Information 2. [file 41598_2025_24730_MOESM2_ESM.jpg]
